# Modulating Optoelectronic and Elastic Properties of Anatase TiO_2_ for Photoelectrochemical Water Splitting

**DOI:** 10.3390/molecules28073252

**Published:** 2023-04-05

**Authors:** Akbar Hussain, Abdur Rauf, Ejaz Ahmed, Muhammad Saleem Khan, Shabeer Ahmad Mian, Joonkyung Jang

**Affiliations:** 1 Department of Physics, University of Peshawar, Peshawar 25120, Pakistan; 2 Department of Physics, Abdul Wali Khan University, Mardan 23200, Pakistan; 3 Department of Chemical Engineering, NFC Institute of Engineering & Technology, Multan 60000, Pakistan; 4 Department of Nano Energy Engineering, Pusan National University, Busan 46241, Republic of Korea

**Keywords:** optoelectronic, elastic moduli, transition metals, DFT, NHE, water splitting

## Abstract

Titanium dioxide (TiO_2_) has been investigated for solar-energy-driven photoelectrical water splitting due to its suitable band gap, abundance, cost savings, environmental friendliness, and chemical stability. However, its poor conductivity, weak light absorption, and large indirect bandgap (3.2 eV) has limited its application in water splitting. In this study, we precisely targeted these limitations using first-principle techniques. TiO_2_ only absorbs near-ultraviolet radiation; therefore, the substitution (2.1%) of Ag, Fe, and Co in TiO_2_ significantly altered its physical properties and shifted the bandgap from the ultraviolet to the visible region. Cobalt (Co) substitution in TiO_2_ resulted in high absorption and photoconductivity and a low bandgap energy suitable for the reduction in water without the need for external energy. The calculated elastic properties of Co-doped TiO_2_ indicate the ductile nature of the material with a strong average bond strength. Co-doped TiO_2_ exhibited fewer microcracks with a mechanically stable composition.

## 1. Introduction

Overcoming the energy crisis without affecting the environment is a crucial challenge for scientists and engineers in the near future. For this purpose, various techniques have been proposed, and the utilization of absorbed sunlight energy through photo/electrocatalysis is an especially promising solution. Honda and Fujishima achieved water splitting using a TiO_2_-based photo/electrocatalyst. TiO_2_ exists in three phases: anatase, brookite, and rutile [1]. They are all used in photo-electrochemical water-splitting applications [2]. Among them, TiO_2_ anatase has been hailed as one of the most promising candidates due to its indirect bandgap [3]. In contrast, brookite and rutile exhibited a direct bandgap of 3.0 and 3.5 eV [2,4,5]. Photo-excited electrons have a longer lifetime in indirect bandgap semiconductors than in direct bandgap semiconductors [2,6] because photo-excited electrons cannot migrate directly from the conduction band (CB) to the valence band (VB) [2], decreasing the charge carrier recombination rate. Additionally, due to the rapid migration of charge carriers [2], TiO_2_ anatase had a lower charge-carrier-recombination rate and the smallest average effective mass [7] of photo-generated electrons, as compared to the brookite and rutile phases; therefore, the superior photo-electrochemical water-splitting properties exhibited by TiO_2_ anatase were not surprising.

Semiconductors such as hematite Fe_2_O_3_ [8,9,10], tungsten trioxide WO_3_ [11,12,13], and TiO_2_ [14,15,16], as well as perovskites including ABX_3_ [17], SrTiO_3_ [18], metal oxides [19], and metal sulfides [20], have been used for this purpose. However, due to its low cost of production [21], easy availability, non-toxicity [22], and thermodynamic stability, TiO_2_ anatase has been investigated meticulously [23,24]. Modern industrialization, technological development, and the rapidly growing global population have caused higher energy consumption, which has resulted in severe environmental pollution due to the burning of hydrocarbons. Clean energy is required to achieve a green environment. Hydrogen is a renewable source, and water is available in an unlimited supply. Photo-electrochemical water splitting resulting in hydrogen fuel and oxygen gas by utilizing sunlight through the application of photo-electrode materials has considerable technological potential [25]. The nature of the delocalized photo-excited holes and electrons, which are generated because of the absorption of light photons of appropriate energy, could play a vital role in the application of water for redox reactions to produce hydrogen and oxygen. The reaction of water with the photo-anode surface occurs in the following manner.

(1) $$H_{2}O+2e^{+}\;\to \;2H^{+}+\frac{1}{2}O_{2}$$

After the oxidation of water, the electrons are transferred to the cathode using an external circuit, while H^+^ moves toward the cathode within the electrolyte, where H^+^ and e^−^ react in the following manner:

2H^+^ + 2e^−^ → H_2_ (2)

Pure TiO_2_ anatase absorbs only ultraviolet light (3–4% of the solar spectrum) due to its wide (3.2 eV) bandgap [3]. Furthermore, it is extremely easy for photo-generated holes and electrons to recombine into pure TiO_2_ anatase, which reduces the photo-electrochemical water-splitting phenomena [26]. Therefore, only two problems have been encountered during this enhanced photo-electrochemical water splitting: (1) a shrinking of the bandgap and (2) a reduction in the charge-carrier-recombination rate. Various existing techniques, such as doping, surface modification, and the construction of heterojunctions, have been proposed to overcome these flaws [27,28]. Among them, doping techniques have significantly improved the optical, electrical, and photocatalytic performance of TiO_2_. Using the developed methodology, the aforementioned issues have been addressed with nonmetallic (N, S, P, B, and C [29,30]) and metallic (Fe, Mo, Au, Rh, and Cu) dopants [31]. Giovanni et al. investigated the N-doping effect on anatase surfaces using first-principle calculations and realized enhanced light absorption because of the creation of oxygen vacancy [23]. Xiaoye et al. studied metal-doped anatase and reported that Cu-doping significantly enhanced its photocatalytic activity in PEC applications. Furthermore, Anindita et al. performed thermoelectric mismatching and bandgap calculations for anatase thin films [24]. Illyas et al. comprehensively studied the optical and electrical properties of TiO_2_ rutile and found that Co-doped TiO_2_ rutile absorbed a large spectrum of solar light. To obtain sufficient knowledge regarding the interatomic and other solid-state phenomena, understanding the elastic properties are crucial. Due to the super-hard nature of TiO_2_ anatase, it has become a prevalent interest of researchers; therefore, the calculations of the mechanical properties are as vital as those of the electronic and optical properties [32].

Herein, the anatase phase of TiO_2_ was comprehensively studied using density-functional-theory (DFT) calculations for PEC water-splitting applications. We thoroughly investigated the impact of Ag, Fe, and Co dopants on the electronic and optical properties of pristine anatase using a DFT + U approach. In addition, the elastic constants (Cij), as well as the shear (G) and bulk (B) moduli, were probed for multifarious fundamental solid-state physical characteristics, including Young’s modulus (Y), the Poisson coefficient (υ), and anisotropy (A^U^).

### Computational Methods and Details

We performed our calculations using DFT with the base variable as the density of electrons, instead of the wave function implemented in SIESTA [33,34,35]. The functionals of the exchange correlation GGA with exchange correlation of revised the Pardew–Burke–Ernzerhof (RPBE) [36,37] for geometry relation were used. A 2 × 2 × 1 supercell was modeled with 48 atoms to perform the calculations. Furthermore, 6 × 6 × 6 k-points were used for the optical properties, and 3 × 3 × 1 k-points were used for the geometry optimization. A cutoff energy of 200 Ry was selected for all structural optimizations as well as for electronic and optical property calculations. Due to the presence of the strongly correlated d-orbital of Ti, we used an effective U-value of 3.5 eV of the Hubbard model (RPBE+U). Herein, a pseudo-atomic orbital (PAO) basis set with a double-zeta potential (DZP) was assigned to all the atoms in the configuration. In addition, we performed all the calculations using the GGA+U method. Elastic property calculations were carried out using the CASTEP code by considering the GGA proposed by PBE with a 340 eV cut-off plane wave basis set. Additionally, all optical properties’ calculations, including absorption coefficients, reflectivity, and energy loss functions were obtained from [38,39,40] in the interval from 250 to 700 nm.

## 2. Results and Discussion

### 2.1. Geometrical Analysis

**Figure 1:**
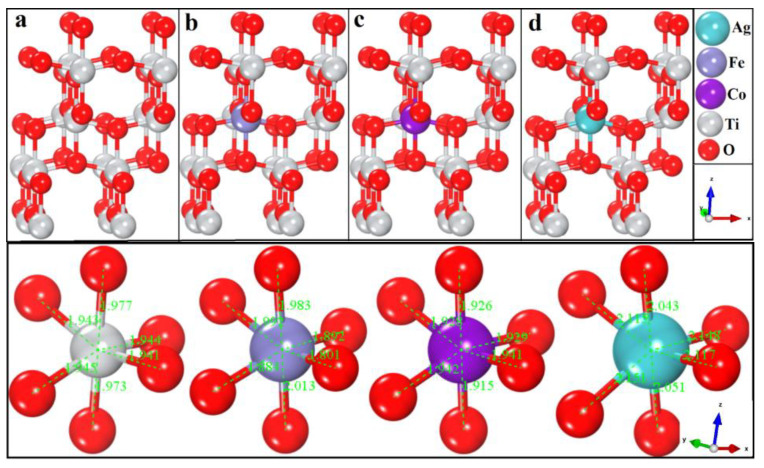
Relaxed geometries of (**a**) TiO_2_, (**b**) TiO_2_/Fe, (**c**) TiO_2_/Co, and (**d**) TiO_2_/Ag.

The effects of doping (Ag, Co, and Fe) on the tetragonal structure of TiO_2_ anatase were explored using DFT. The defects produced by the dopants in the host supercell matrix were simulated. The defect geometries were obtained by substituting the Ti atoms with each dopant individually. The optimized geometries of the doped and pure TiO_2_ anatases are given in Figure 1.

**Figure 2:**
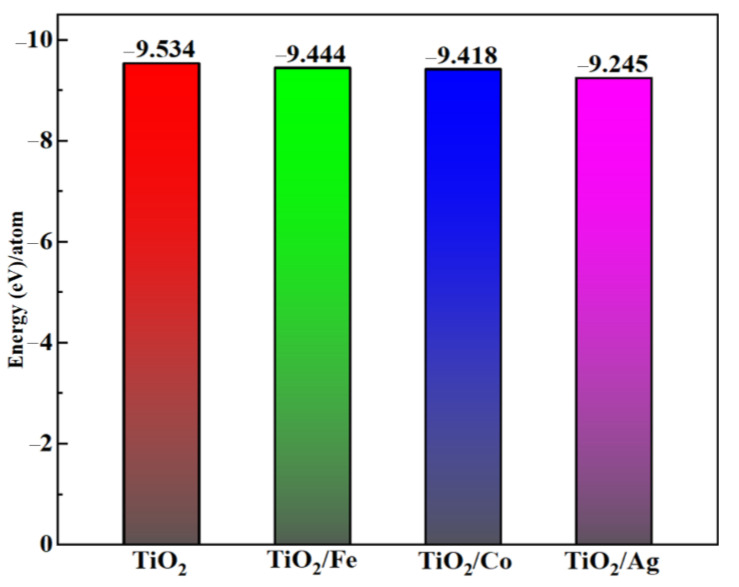
Formation energies per atom of pure and doped TiO_2_.

The lattice vectors estimated at the lowest energies are listed in Table 1. The bond lengths of Ti and each dopant with O atoms were calculated and found to be in good agreement with the experimental data and, therefore, deemed favorable for further study. The incorporation of Fe and Co resulted in decreased bond lengths, which caused the cell volume to decrease, as shown in Table 1. Furthermore, the Ag dopant expanded the cell volume due to the long Ag–O bond length. The thermodynamic stability of each doped TiO_2_ sample was calculated using the formation energy of pristine and doped TiO_2_, using the following equations:

(3) $$E_{F}=\frac{1}{N}\left(E_{T}-\sum_{i=\mathrm{Ti},O,\mathrm{Ag},\mathrm{Co},\mathrm{Fe}}n_{i}E_{i}\right)$$

where *E_T_* represents the total energy of the preferred system consisting of *N* atoms. The variable *E_i_* demonstrates the total energy of an isolated *i* (Ti, O, Ag, Co, Fe) atom, and *n_i_* is the total number of a specific atom *i* per unit cell. Our estimated formation energy for pristine and Fe-, Co-, and Ag-doped TiO_2_ were −9.534, −9.444, −9.418, and −9.245 eV, respectively. Higher negative values of the formation energy indicated their favorable and easy synthesis in the laboratory; this was consistent with the data in the literature. The comparative formation energies of doped and pristine TiO_2_ are shown in Figure 2.

### 2.2. Electronic Properties

**Figure 3:**
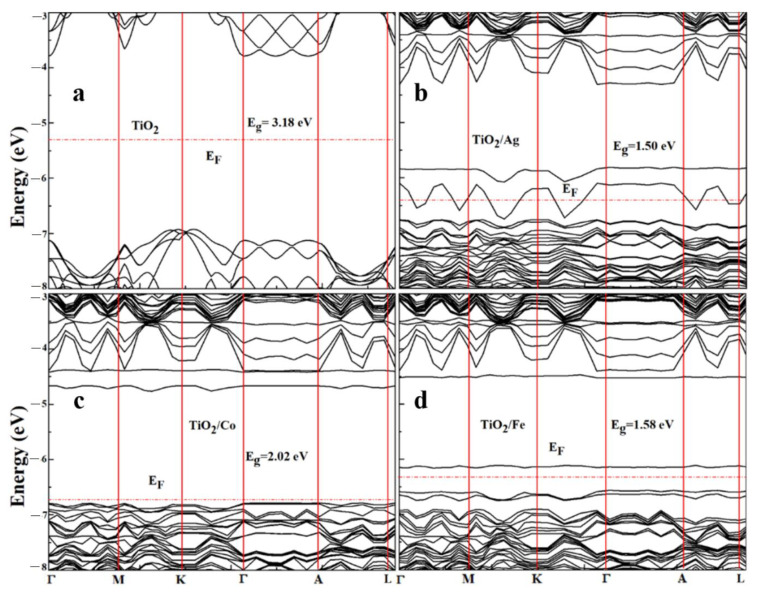
Band structure of (**a**) pristine and (**b**) Ag-, (**c**) Co-, and (**d**) Fe-doped TiO_2_.

To understand the electronic and optical properties of the pristine and doped compositions, an investigation of the electronic band structure was of prime importance. The band structures of pristine and doped TiO_2_ in the SIESTA code were calculated using the GGA+U approximation. All band-structure calculations were performed using high-symmetry points along special lines in the k-space. Figure 3 depicts the band structures of the pristine and doped systems. As shown in Figure 3, TiO_2_ was an indirect bandgap (3.18 eV) semiconductor, which was in good agreement with the experimental results [41].

Furthermore, TiO_2_/Ag, TiO_2_/Co, and TiO_2_/Fe showed direct bandgaps of 1.50, 2.02, and 1.58 eV, respectively, along the high-symmetry directions of the Brillouin zones and were in good agreement with another work, as shown in Table 2 [43,44].

The incorporation of the dopants (Ag, Co, and Fe) in TiO_2_ caused the bandgaps for each compound to shrink, which resulted in the maximum absorption of the solar spectrum, tumbling the rate of recombination and increasing the generation of electron–hole pairs for efficient PEC activity. This reduction occurred in the bandgaps because of the generation of novel energy states near the Fermi level.

**Figure 4:**
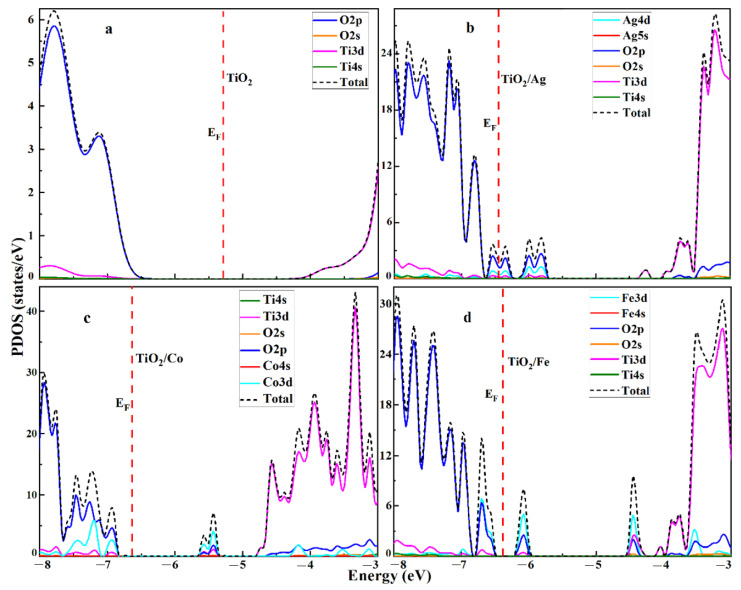
Projected density of states of (**a**) pristine and (**b**) Ag-, (**c**) Co-, and (**d**) Fe-doped TiO_2_.

To unravel the composition of the valence and conduction bands, the projected density of the states (PDOS) for both the pristine and doped TiO_2_ were calculated, as portrayed in Figure 4. For comparison, the PDOS of pristine TiO_2_ is also shown in Figure 4a, in which the valance band edge was mostly composed of O-2p states, whereas the conduction band edge was attributed to the unoccupied Ti-3d atomic orbitals. In the case of the Ag dopant, the new states generated by the Ag-3d atomic orbital occurred in the valence-band edge with the overlapping of O-2p states. Similarly, when a Ti atom was substituted by a Co atom, it induces new states of the Co-3d orbitals mixed in with the O-2p and Ti-3d states in the valence band. For the Fe substitution of Ti, the Fe-3d states occurred in the valence band, causing a redshift of absorption and enhancing the photo-response in the visible region. Conclusively, the substitutional impurities in titania caused the shrinkage of the bandgap due to the creation of novel states in the mid-gap, thus reducing the energy barrier in the photon-absorption process, which was the primary condition for high-efficiency photo-anodes in the PEC process.

**Figure 5:**
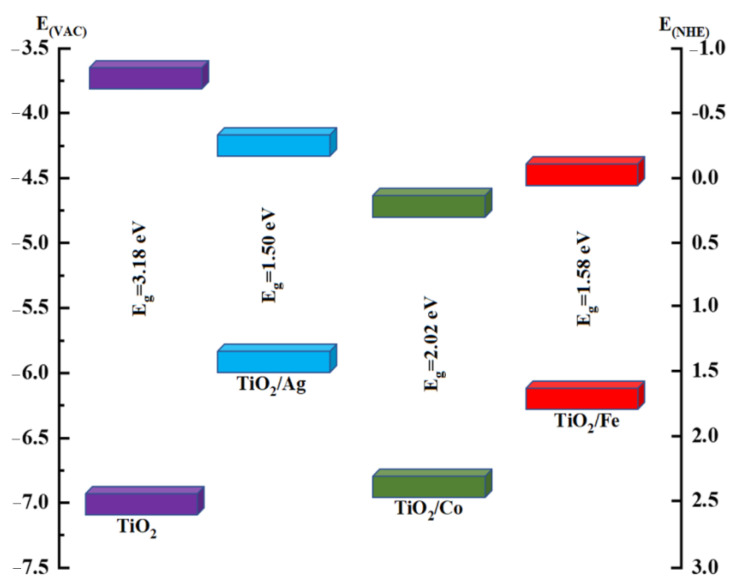
Band-edge comparisons of pure and doped TiO_2_ anatase in electron volts. The energy scale is represented in (eV) using either the normal hydrogen-electrode potential (NHE) or the vacuum level as a reference.

The generation of H_2_ via water splitting has been of great importance [46]. To induce water splitting without external energy, the positions of the valence and conduction bands had to be more positive and negative than the oxidation and reduction potentials of the water, respectively. The oxidation potential of water is +1.23 V while its reduction potential is +0 V vs. NHE [47]. In photocatalytic processes, charge separation is a vital factor. If successful charge separation occurs, these charges move to the semiconductor surface and participate in the oxidation and reduction processes. Figure 5 illustrates the energies of the VBM and the CBM of pristine and doped anatase for efficient photocatalytic properties [15]. However, Figure 5 shows that the CBM of anatase was more negative than the water oxidation potential, whereas the VBM edge was more positive, indicating its superiority for PEC activity.

The Ag-doped anatase had a suitable conduction-band position for the water reduction reaction, whereas Co-doped anatase had a suitable valence-band position for the water oxidation reaction. Specifically, all dopants significantly improved the VBM position, representing better and promising oxidation reactions for the water-splitting half-reaction. The shift in the CBM and the VBM occurred because of the transition-metal dopants, which contributed to the new energy states.

#### 2.2.1. Real and Imaginary Parts of Dielectric Functions

**Figure 6:**
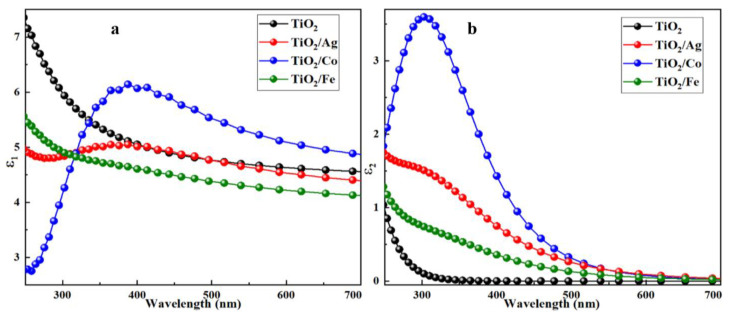
(**a**) Real and (**b**) imaginary parts of the dielectric function for pure and transition-metal-doped TiO_2_.

Dielectric functions define the electronic properties of materials under the effect of incident radiation; they are given by ε(ω) = ε_1_(ω) + i ε_2_(ω). The real part “ε_1_(ω)” demonstrates the material polarizability, whereas the imaginary part ε_2_(ω) describes the electronic absorption of the material when illuminated by a certain incident photon energy. The calculated values of the real and imaginary parts of the pristine and doped TiO_2_ are shown in Figure 6. For pristine TiO_2_, ε_1_ had a peak value of 5.11 at 387 nm, and the incorporation of the dopants increased this value up to 6.14 at the same wavelength, suggesting the maximum electronic polarization for energy storage devices. Among all the dopants, Co exhibited a high polarizability near 387 nm, as shown in Figure 6a. In Figure 6b, the imaginary part ε_2_ exhibited a first peak at 317 nm for pristine TiO_2_. The first edge of each material was associated with the fundamental bandgap E_g_, which represents the transition between the valence-band maximum (VBM) and the conduction-band minimum (CBM). In the case of pristine TiO_2_, the transition of electrons occurred from the O-2p-occupied state (VBM) to the Ti3d unoccupied state (CBM) near the Fermi level in the ultraviolet region, suggesting a higher energy loss than that of the doped TiO_2_. Furthermore, for the Co-doped anatase, the peak value was observed at 300 nm. In general, the substitutional doping improved the real and imaginary parts of the dielectric functions in the overall solar spectrum, with considerable advancement in the visible region.

#### 2.2.2. Optical Absorption and Conductivity

**Figure 7:**
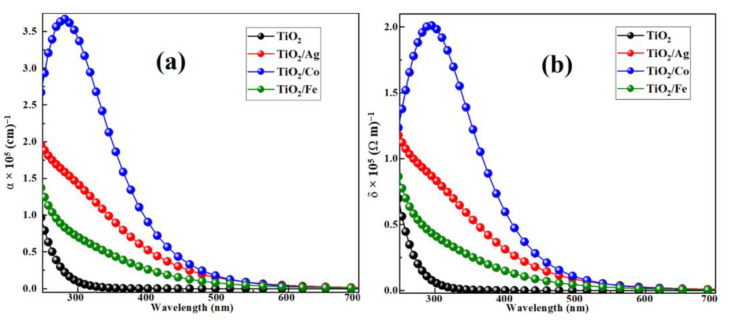
(**a**) Absorption coefficient and (**b**) optical conductivity of pure and metal doped TiO_2_ anatase.

To describe the effect of dopants (Ag, Co, and Fe) on the optical absorption and conductivity, we first calculated the two parameters for pristine and doped TiO_2_, as portrayed in Figure 7. Pristine TiO_2_ had a wide bandgap (3.18 eV) and could only absorb ultraviolet light [42]. The substitutional doping significantly improved the absorption peak in the visible region, showing much better and more promising material properties for photo-electrochemical water-splitting applications. The improvements in the absorption edge, as shown in Figure 7a, were attributed to the narrow bandgap of the pristine TiO_2_, and the existence of the new mediator energy states between the VBM and the CBM had decreased the electronic excitation energy needed. Such states were capable of absorbing low-energy photons and generating electron–hole pairs, both of which increased the photoconversion efficiency of the materials. Our computed optical absorption properties for iron (Fe)-doped TiO_2_ were consistent with theoretical and experimental data [48]. Among all the dopants, Co showed a substantially larger absorption than the other dopants due to the high density of the occupied and unoccupied states, which utilized low-energy photons for the transition from the VBM to the CBM.

In addition, the enhancements of the optical conductivity played a vital role in water splitting and opto-electronic devices, which depend solely on the absorption and refractive index. If a material absorbs greater photon energy than its bandgap, electron–hole pair generation occurs. These pairs move freely in the crystal, which induces optical conductivity. Optical conductivity does not contribute to electrical conductivity because of electronic charge neutrality [49]. For semiconductors and insulators, the electrical conductivity has always been negligible; however, the optical conductivity had been finite due to the optical bandgap. Figure 7b illustrates that the incorporation of the dopants in TiO_2_ increased its conductivity, with the largest (by a considerable margin) peak in Co-doped anatase. This enhancement would increase the water reduction reactions due to a redshift in the bandgap energy, which would result in the maximum absorption of the solar spectrum. Optical conductivity followed the same trend as the absorption coefficient.

**Figure 8:**
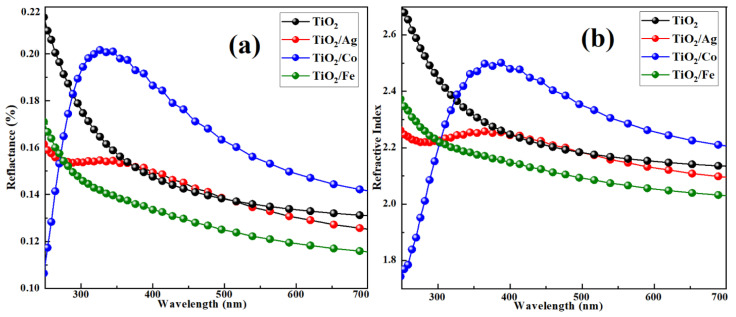
(**a**) Reflectance and (**b**) refractive index for pure and doped TiO_2_ anatase.

The calculated reflectance and refractive index for both pristine and transition-metal-doped TiO_2_ as a function of the wavelength is shown in Figure 8. The refractive index determines the speed of light propagation in a medium. For a given material, the maximum value of the refractive index indicates that the light slowly propagates within the material; the opposite is true for the minimum refractive index. For doped TiO_2_, the refractive index showed a decreasing trend at higher wavelengths and an increasing trend at lower wavelengths. The decreasing trend was due to the optical dispersion of the materials [50]. Cobalt-doped TiO_2_ exhibited a maximum refractive index value of 2.51 at *n* = 380 nm, as portrayed in Figure 8.

Figure 8a demonstrates the estimated values of the reflectance for the materials. The reflectivity for Co-doped TiO_2_ exhibited a maximum value of approximately 20%. In addition, the pristine and Fe- and Ag-doped TiO_2_ showed < 15% reflectivity in the visible range, suggesting their suitability in antireflective-coating applications [51]. All the compositions with weak absorption and conduction exhibited stronger reflectance.

**Figure 9:**
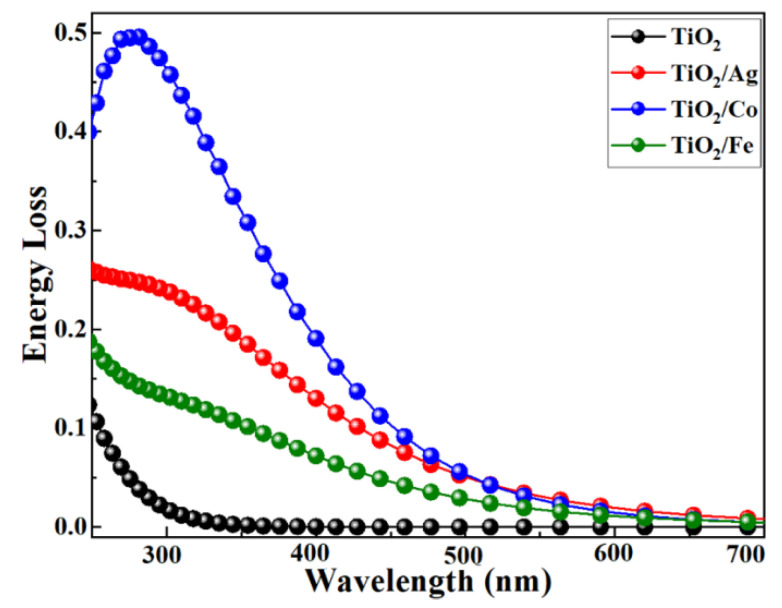
Energy loss vs. wavelength for pristine and transition-metal-doped TiO_2_.

The loss spectrum was another optical parameter that revealed the spectrum loss during its propagation through the medium. When electrons moved inside the solids, inelastic scattering occurred, which was related to the energy-loss function. Figure 9 displays the energy-loss spectrum as a function of the wavelength from the VBM to the CBM. In addition, the loss spectrum indicated the trailing edges of the reflection spectrum. The highest energy-loss peak characterized the plasma frequency and related resonance. Furthermore, Co-doped TiO_2_ showed a sharp decrease, as compared to the other dopants, indicating a rapid decrease in reflectivity.

### 2.3. Elastic Properties

The crystal responses to the external parameters, such as strain and pressure, were governed by the elastic characteristics of the material, which included its mechanical stability, the modes of the phonons, and other solid-state phenomena. To understand the mechanical properties of any solid, the elastic constants (C_ij_) listed in Table 3 were of great importance; they could be obtained by the Taylor-series expansion [52].

Due to the symmetry, each crystal had its own specific elastic constant. As TiO_2_ (anatase) exists in tetragonal symmetry, there were six independent elastic constants: C_11_, C_12_, C_13_, C_33_, C_44_, and C_66_. These elastic constants correlated the stress (σ) and strain (ε) tensors using Hooke’s law. Table 4 depicts the estimated values of the elastic moduli, could be derived from the Voigt–Reuss–Hill approximations and were in good agreement with existing theoretical and experimental results [54]. The mechanical stability was determined by the strain energy, which had to be positive for any elastic deformation. For tetragonal symmetry, the crystal had to fulfill the following criteria: C_11_ > 0, C_44_ > 0, C_66_ > 0, C_11_ − C_12_ > 0, and C_11_C_33_ + C_12_C_33_ − 2C_13_^2^ > 0. Our findings revealed that all the conditions were successfully satisfied, and that all the materials were mechanically stable.

Bulk modulus (B) is the capability of the material volume to resist elastic deformation. It indicates the binding energy and average bond strength of atoms in a crystal [52]. Table 3 shows the bulk modulus of pristine and Co-doped TiO_2_ with the values of 192.736 and 176.590 GPa, respectively, indicating a high average bond strength between Ti and O [55].

Figure 4 shows that the structural properties were attributed to the O-2p and Ti-3d states, indicating ionic and partially covalent bonds. This spatial arrangement made it arduous to modify the atomic displacement through the action of an external force. Meanwhile, the isoionic bond and covalent bond directionality made dislocation almost impossible and created obstacles to dislocation propagation for the surrounding grains. At room temperature, the pristine and doped TiO_2_ were sensitive to deformation. Under the influence of an external force, if breaking occurred in the structure, it indicated brittleness, poor toughness, and a relatively low shear modulus, as shown in Table 4.

In addition, Poisson’s ratio (υ) governs material brittleness and ductility, along with the expansion capability in the direction normal of the compression. A material with a Poisson’s ratio less than 0.33 was considered to be fragile; otherwise, it was considered ductile [39]. Pristine TiO_2_ has a higher Poisson’s ratio than Co-doped TiO_2_, indicating a higher ductility. Moreover, a Pugh’s ratio (B/G) greater than 1.75 suggested a ductile nature, and our estimated results also indicated a ductile nature in both systems, suggesting consistency with Poisson’s ratio [39].

To understand the directional dependence of physical properties, anisotropy (A^U^) is very important. The null value of anisotropy indicates the isotropic nature of the material. Furthermore, it also determines the nature of the microcracks and the phase stability of crystal structures. A higher anisotropy value indicates microcracks and stress concentration. Our calculation revealed that the incorporation of Co produced fewer microcracks than found in pristine TiO_2_. Moreover, the Poisson’s ratio and anisotropy values were in good agreement with the literature data and are listed in Table 4. The elastic properties were related to the thermal properties, including the Grüneisen parameter and Debye temperature. The corresponding mechanical properties were promising for energy-associated applications [56].

## 3. Conclusions

The optoelectronic properties of pristine and doped TiO_2_ were meticulously analyzed for PEC water splitting using DFT simulations. It was observed that a 2.1% incorporation of each dopant accentuated the optical absorption and the charge transport capabilities in a broad range of the solar spectrum. The calculated bandgap was reduced to 1.50 eV, 2.02 eV, and 1.58 eV in the doped variants of silver, cobalt, and iron, respectively, due to the generation of novel states and the sensitization of the materials to the visible and infrared region, indicating promising PEC water-splitting activity. Furthermore, a more negative CBM and a more positive VBM, with respect to the water oxidation and reduction, respectively, made the materials more efficacious for water splitting. In addition, the enhancement in the optical absorption due to substitutional doping increased the carrier concentration, which overcame the electron–hole pair recombination. Because of the suitable bandgap of TiO_2_/Co, we estimated its elastic properties and observed that it had the highest average bond strength, as well as high ductility. Our calculations also revealed mechanically stable configurations, fewer microcracks, and lower stress concentrations in Co-doped TiO_2_, as compared to pristine TiO_2_. In general, substitutional doping significantly altered the optical conductivity, the dielectric function, the reflectance, and the refractive index of the materials, suggesting their technological potential in PEC water splitting.

## Figures and Tables

**Table 1:** Lattice parameters, volume, and c/a of pristine and doped TiO_2_ anatase.

Type	a (Å)	c(Å)	c/a	V (Å)^3^
TiO_2_-Pure DFT [41] Experiment [42]	3.791 3.737, 3.741 3.785	9.510 9.981, 9.964 9.514	2.51 2.57, 2.66 2.51	549.537
TiO_2_/Ag DFT [43]	3.821 3.905	9.588 9.825	2.51 2.52	559.621
TiO_2_/Co	3.802	9.447	2.48	545.971
TiO_2_/Fe DFT [44]	3.785 3.771	9.496 9.489	2.51 2.49	545.984

**Table 2:** Comparative bandgaps of pristine and doped TiO_2_ anatase.

Materials	This Work	Other DFT	Experimental
TiO_2_	3.18 eV	3.3 eV [45]	3.2 eV [42]
TiO_2_/Ag	1.50 eV	0.9 eV [43]	---
TiO_2_/Co	2.02 eV	---	---
TiO_2_/Fe	1.58 eV	1.74 eV [44]	---

**Table 3:** Elastic constants (C_ij_; GPa) for pure and Co-doped TiO_2_ anatase comparison data.

Elastic Constants	This Work TiO_2_	Other DFT [39]^a^ [53]^b^	This Work TiO_2_/Co
C_11_	369.140	^a^399.1, ^b^336.5	340.595
C_12_	153.918	^a^167.6, ^b^138.6	155.326
C_13_	153.260	^a^159.9, ^b^136.0	138.609
C_33_	215.389	^a^250.8, ^b^192.1	196.803
C_44_	44.281	^a^70.49, ^b^49.4	60.523
C_66_	58.665	^a^61.28, ^b^58.3	63.446

^a^ Ref. [39]; ^b^ [53].

**Table 4:** Estimated elastic moduli and allied parameters of pristine and Co-doped TiO_2_.

Elastic Properties	Current Work TiO_2_	Other Work TiO_2_ [55]	Current Work Co dope TiO_2_
B_V_	208.283	221.982	193.675
B_R_	192.736	204.210	176.590
B_H_	200.510	213.096	185.132
G_v_	62.327	51.275	66.595
G_R_	55.230	32.085	63.099
G_H_	58.779	41.680	64.847
E_V_	170.023	142.829	179.241
E_R_	151.243	91.466	169.152
E_H_	160.640	117.388	174.203
υ_V_	0.363	0.393	0.346
υ_R_	0.369	0.425	0.340
υ_H_	0.366	0.408	0.343
B_V_/G_V_	3.342	4.329	2.908
B_R_/G_R_	3.489	6.365	2.799
B_H_/G_H_	3.411	5.113	2.855
A^U^	0.423	0.368	0.374

## Data Availability

Not applicable.

## Sample Availability

Samples of the compounds are available from the authors.
